# Giant cell arteritis with polymyalgia rheumatica on FDG‐PET/CT

**DOI:** 10.1002/ccr3.994

**Published:** 2017-05-21

**Authors:** Akira Baba, Kimiichi Uno, Yumi Okuyama, Yohei Munetomo, Shintaro Nakajima, Kennosuke Mizushina, Hideto Kameda

**Affiliations:** ^1^ Department of RadiologyTokyo Dental College Ichikawa General HospitalChibaJapan; ^2^ Gaien Higashi ClinicTokyoJapan; ^3^ Department of RheumatologyToho University Medical Center Ohashi HospitalTokyoJapan

**Keywords:** FDG‐PET/CT, giant cell arteritis, polymyalgia rheumatica

## Abstract

If there is no pain in the temporal artery, the diagnosis of giant cell arteritis (GCA) may be delayed and blindness may occur. Therefore, FDG‐PET/CT is important as a modality for diagnosis of GCA. When GCA is suspected and F‐18 FDG‐PET/CT is performed, it is worthwhile to pay attention to shoulder and hip joints as polymyalgia rheumatica commonly presents with GCA.

An 80‐year‐old woman presented with a two‐month history of general malaise and bilateral shoulder and hip pain with morning stiffness. Her past history was pertinent with chronic subdural hematoma. Laboratory investigations revealed increased C‐reactive protein level (5.67 mg/dL) only and no other abnormal results. Contrast‐enhanced CT images revealed wall thickening of aorta, suggesting the aortitis. F‐18 FDG‐PET/CT confirmed the diagnosis of giant cell arteritis (GCA), with increased FDG uptake in the wall of aorta and bilateral subclavian arteries (Figs 1 and 2, arrows). It also showed the increased uptake in shoulders and hip joints (Figs 1 and 2, arrowheads), which was compatible with the diagnosis of polymyalgia rheumatica (PMR). She underwent oral steroid and methotrexate treatment, and both symptoms and radiological signs improved. If patients presented without headache or scalp tenderness, like our case, clinicians may not suspect GCA, and visual loss may occur because of the delayed diagnosis 1. Therefore, FDG‐PET/CT is important as a tool for diagnosis of GCA. It is known that patients with PMR are often accompanied with GCA 2. In our case, PET/CT successfully captured the simultaneous active inflammation at typical sites for GCA and PMR: major arteries and proximal joints, including hip joints. F‐18 FDG‐PET/CT is useful to diagnose both GCA and PMR, and it is worthy to check proximal joints along with major blood vessels 2, 3.

**Figure 1 and 2:**
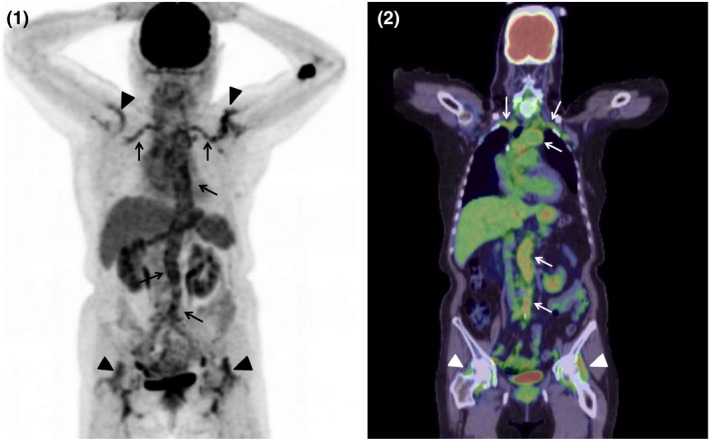
F‐18 FDG‐PET/CT showed increased FDG uptake in the wall of aorta, bilateral subclavian arteries (1 and 2, arrows), and shoulders and hip joints (1 and 2, arrowheads).

## Authorship

AB: drafted the article. All authors: participated in critical review and revision of the article, gave the final approval of the article, and have accountability for all aspects of the work.

## Conflict of Interest

None declared.
